# Body language in the brain: constructing meaning from expressive movement

**DOI:** 10.3389/fnhum.2015.00450

**Published:** 2015-08-21

**Authors:** Christine M. Tipper, Giulia Signorini, Scott T. Grafton

**Affiliations:** ^1^Department of Psychiatry, University of British ColumbiaVancouver, BC, Canada; ^2^Mental Health and Integrated Neurobehavioral Development Research Core, Child and Family Research InstituteVancouver, BC, Canada; ^3^Psychiatric Epidemiology and Evaluation Unit, Saint John of God Clinical Research CenterBrescia, Italy; ^4^Department of Psychological and Brain Sciences, University of CaliforniaSanta Barbara, CA, USA

**Keywords:** action observation, dance, social neuroscience, fMRI, repetition suppression, predictive coding

## Abstract

This fMRI study investigated neural systems that interpret body language—the meaningful emotive expressions conveyed by body movement. Participants watched videos of performers engaged in modern dance or pantomime that conveyed specific themes such as hope, agony, lust, or exhaustion. We tested whether the meaning of an affectively laden performance was decoded in localized brain substrates as a distinct property of action separable from other superficial features, such as choreography, kinematics, performer, and low-level visual stimuli. A repetition suppression (RS) procedure was used to identify brain regions that decoded the meaningful affective state of a performer, as evidenced by decreased activity when emotive themes were repeated in successive performances. Because the theme was the only feature repeated across video clips that were otherwise entirely different, the occurrence of RS identified brain substrates that differentially coded the specific meaning of expressive performances. RS was observed bilaterally, extending anteriorly along middle and superior temporal gyri into temporal pole, medially into insula, rostrally into inferior orbitofrontal cortex, and caudally into hippocampus and amygdala. Behavioral data on a separate task indicated that interpreting themes from modern dance was more difficult than interpreting pantomime; a result that was also reflected in the fMRI data. There was greater RS in left hemisphere, suggesting that the more abstract metaphors used to express themes in dance compared to pantomime posed a greater challenge to brain substrates directly involved in decoding those themes. We propose that the meaning-sensitive temporal-orbitofrontal regions observed here comprise a superordinate functional module of a known hierarchical action observation network (AON), which is critical to the construction of meaning from expressive movement. The findings are discussed with respect to a predictive coding model of action understanding.

## Introduction

Body language is a powerful form of non-verbal communication providing important clues about the intentions, emotions, and motivations of others. In the course of our everyday lives, we pick up information about what people are thinking and feeling through their body posture, mannerisms, gestures, and the prosody of their movements. This intuitive social awareness is an impressive feat of neural integration; the cumulative result of activity in distributed brain systems specialized for coding a wide range of social information. Reading body language is more than just a matter of perception. It entails not only recognizing and coding socially relevant visual information, but also ascribing *meaning* to those representations.

We know a great deal about brain systems involved in the perception of facial expressions, eye movements, body movement, hand gestures, and goal directed actions, as well as those mediating affective, decision, and motor responses to social stimuli. What is still missing is an understanding of how the brain “reads” body language. Beyond the decoding of body motion, what are the brain substrates directly involved in extracting meaning from affectively laden body expressions? The brain has several functionally specialized structures and systems for processing socially relevant perceptual information. A subcortical pulvinar-superior colliculus-amygdala-striatal circuit mediates reflex-like perception of emotion from body posture, particularly fear, and activates commensurate reflexive motor responses (Dean et al., 1989; Cardinal et al., 2002; Sah et al., 2003; de Gelder and Hadjikhani, 2006). A region of the occipital cortex known as the extrastriate body area (EBA) is sensitive to bodily form (Bonda et al., 1996; Hadjikhani and de Gelder, 2003; Astafiev et al., 2004; Peelen and Downing, 2005; Urgesi et al., 2006). The fusiform gyrus of the ventral occipital and temporal lobes has a critical role in processing faces and facial expressions (McCarthy et al., 1997; Hoffman and Haxby, 2000; Haxby et al., 2002). Posterior superior temporal sulcus is involved in perceiving the motion of biological forms in particular (Allison et al., 2000; Pelphrey et al., 2005). Somatosensory, ventromedial prefrontal, premotor, and insular cortex contribute to one's own embodied awareness of perceived emotional states (Adolphs et al., 2000; Damasio et al., 2000). Visuomotor processing in a functional brain network known as the action observation network (AON) codes observed action in distinct functional modules that together link the perception of action and emotional body language with ongoing behavioral goals and the formation of adaptive reflexes, decisions, and motor behaviors (Grafton et al., 1996; Rizzolatti et al., 1996b, 2001; Hari et al., 1998; Fadiga et al., 2000; Buccino et al., 2001; Grézes et al., 2001; Grèzes et al., 2001; Ferrari et al., 2003; Zentgraf et al., 2005; Bertenthal et al., 2006; de Gelder, 2006; Frey and Gerry, 2006; Ulloa and Pineda, 2007). Given all we know about how bodies, faces, emotions, and actions are perceived, one might expect a clear consensus on how meaning is derived from these percepts. Perhaps surprisingly, while we know these systems are crucial to integrating perceptual information with affective and motor responses, how the brain deciphers meaning based on body movement remains unknown. The focus of this investigation was to identify brain substrates that decode meaning from body movement, as evidenced by meaning-specific neural processing that differentiates body movements conveying distinct expressions.

To identify brain substrates sensitive to the meaningful emotive state of an actor conveyed through body movement, we used repetition suppression (RS) fMRI. This technique identifies regions of the brain that code for a particular stimulus dimension (e.g., shape) by revealing substrates that have different patterns of neural activity in response to different attributes of that dimension (e.g., circle, square, triangle; Grill-Spector et al., 2006). When a particular attribute is repeated, synaptic activity and the associated blood oxygen level-dependent (BOLD) response decreases in voxels containing neuronal assemblies that code that attribute (Wiggs and Martin, 1998; Grill-Spector and Malach, 2001). We have used this method previously to show that various properties of an action such as movement kinematics, object goal, outcome, and context-appropriateness of action mechanics are uniquely coded by different neural substrates within a parietal-frontal action observation network (AON; Hamilton and Grafton, 2006, 2007, 2008; Ortigue et al., 2010). Here, we applied RS-fMRI to identify brain areas in which activity decreased when the meaningful emotive theme of an expressive performance was repeated between trials. The results demonstrate a novel coding function of the AON—decoding meaning from body language.

**Figure 1:**
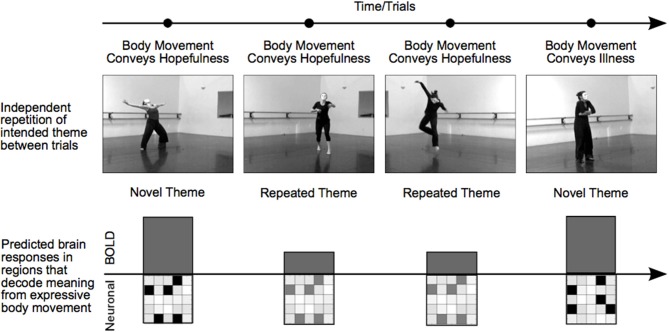
**Manipulating trial sequence to induce RS in brain regions that decode body language**. The order of video presentation was controlled such that themes depicted in consecutive videos were either novel or repeated. Each consecutive video clip was unique; repeated themes were always portrayed by different dancers, different camera angles, or both. Thus, RS for repeated themes was not the result of low-level visual features, but rather identified brain areas that were sensitive to the specific meaningful theme conveyed by a performance. In brain regions showing RS, a particular affective theme—hope, for example—will evoke a particular pattern of neural activity. A novel theme on the subsequent trial—illness, for instance—will trigger a different but equally strong pattern of neural activity in distinct cell assemblies, resulting in an equivalent BOLD response. In contrast, a repetition of the hopefulness theme on the subsequent trial will trigger activity in the same neural assemblies as the first trial, but to a lesser extent, resulting in a reduced BOLD response for repeated themes. In this way, regions showing RS reveal regions that support distinct patterns of neural activity in response to different themes.

Working with a group of professional dancers, we produced a set of video clips in which performers intentionally expressed a particular meaningful theme either through dance or pantomime. Typical themes consisted of expressions of hope, agony, lust, or exhaustion. The experimental manipulation of theme was studied independently of choreography, performer, or camera viewpoint, which allowed us to repeat the meaning of a movement sequence from one trial to another while varying physical movement characteristics and perceptual features. With this RS-fMRI design, a decrease in BOLD activity for repeated relative to novel themes (RS) could not be attributed to specific movements, characteristics of the performer, “low-level” visual features, or the general process of attending to body expressions. Rather, RS revealed brain areas in which specific voxel-wise neural population codes differentiated meaningful expressions based on body movement (Figure 1).

Participants were scanned using fMRI while viewing a series of 10-s video clips depicting modern dance or pantomime performances that conveyed specific meaningful themes. Because each performer had a unique artistic style, the same theme could be portrayed using completely different physical movements. This allowed the repetition of meaning while all other aspects of the physical stimuli varied from trial to trial. We predicted that specific regions of the AON engaged by observing expressive whole body movement would show suppressed BOLD activation for repeated relative to novel themes (RS). Brain regions showing RS would reveal brain substrates directly involved in decoding meaning based on body movement.

The dance and pantomime performances used here conveyed expressive themes through movement, but did not rely on typified, canonical facial expressions to invoke particular affective responses. Rather, meaningful themes were expressed with unique artistic choreography while facial expressions were concealed with a classic white mime's mask. The result was a subtle stimulus set that promoted thoughtful, interpretive viewing that could not elicit reflex-like responses based on prototypical facial expressions. In so doing, the present study shifted the focus away from automatic affective resonance toward a more deliberate ascertainment of meaning from movement.

While dance and pantomime both expressed meaningful emotive themes, the quality of movement and the types of gestures used were different. Pantomime sequences used fairly mundane gestures and natural, everyday movements. Dance sequences used stylized gestures and interpretive, prosodic movements. The critical distinction between these two types of expressive movement is in the degree of abstraction in the metaphors that link movement with meaning (see Morris, 2002 for a detailed discussion of movement metaphors). Pantomime by definition uses gesture to mimic everyday objects, situations, and behavior, and thus relies on relatively concrete movement metaphors. In contrast, dance relies on more abstract movement metaphors that draw on indirect associations between qualities of movement and the emotions and thoughts it evokes in a viewer. We predicted that since dance expresses meaning more abstractly than pantomime, dance sequences would be more difficult to interpret than pantomimed sequences, and would likewise pose a greater challenge to brain processes involved in decoding meaning from movement. Thus, we predicted greater involvement of thematic decoding areas for danced than for pantomimed movement expressions. Greater RS for dance than pantomime could result from dance triggering greater activity upon a first presentation, a greater reduction in activity with a repeated presentation, or some combination of both. Given our prediction that greater RS for dance would be linked to interpretive difficulty, we hypothesized it would be manifested as an increased processing demand resulting in greater initial BOLD activity for novel danced themes.

## Methods

### Participants

Forty-six neurologically healthy, right-handed individuals (30 women, mean age = 24.22 years, range = 19–55 years) provided written informed consent and were paid for their participation. Performers also agreed in writing to allow the use of their images and videos for scientific purposes. The protocol was approved by the Office of Research Human Subjects Committee at the University of California Santa Barbara (UCSB).

### Stimuli

Eight themes were depicted, including four danced themes (happy, hopeful, fearful, and in agony) and four pantomimed themes (in love, relaxed, ill, and exhausted). Performance sequences were choreographed and performed by four professional dancers recruited from the SonneBlauma Danscz Theatre Company (Santa Barbara, California; now called ArtBark International, http://www.artbark.org/). Performers wore expressionless white masks so body language was conveyed though gestural whole-body movement as opposed to facial expressions. To express each theme, performers adopted an affective stance and improvised a short sequence of modern dance choreography (two themes per performer) or pantomime gestures (two themes per performer). Each of the eight themes were performed by two different dancers and recorded from two different camera angles, resulting in four distinct videos representing each theme (32 distinct videos in total; clips available in Supplementary Materials online).

### Behavioral procedure

**Figure 2:**
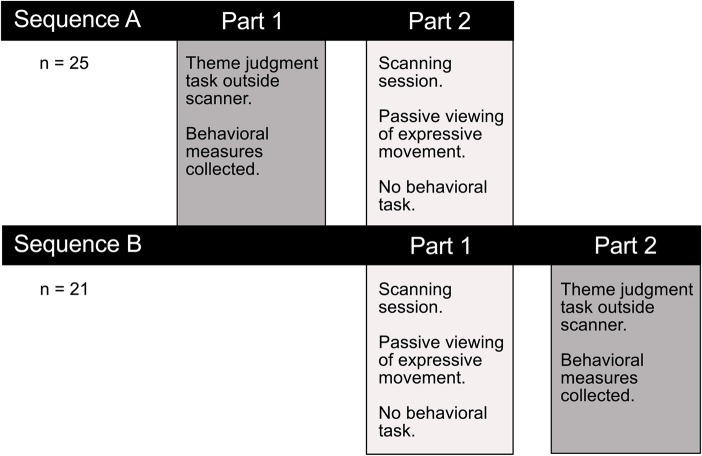
**Experimental testing procedure**. Participants completed a thematic interpretation task outside the scanner, either before or after the imaging session. Performance on this task allowed us to test whether there was a difference in how readily observers interpreted the intended meaning conveyed through dance or pantomime. Any performance differences on this explicit theme judgment task could help interpret the functional significance of observed differences in brain activity associated with passively viewing the two types of movement in the scanner.

In a separate session outside the scanner either before or after fMRI data collection, an interpretation task measured observers' ability to discern the intended meaning of a performance (Figure 2). The interpretation task was carried out in a separate session to avoid confounding movement observation in the scanner with explicit decision-making and overt motor responses. Participants were asked to view each video clip and choose from a list of four options the theme that best corresponded with the movement sequence they had just watched. Responses were made by pressing one of four corresponding buttons on a keyboard. Two behavioral measures were collected to assess how well participants interpreted the intended meaning of expressive performances. Consistency scores reflected the proportion of observers' interpretations that matched the performer's intended expression. Response times indicated the time taken to make interpretive judgments. In order to encourage subjects to use their initial impressions and to avoid over-deliberating, the four response options were previewed briefly immediately prior to video presentation.

For the interpretation task collected outside the scanner, videos were presented and responses collected on a Mac Powerbook G4 laptop programmed using the Psychtoolbox (v. 3.0.8) extension (Brainard, 1997; Pelli and Brainard, 1997) for Mac OSX running under Matlab 7.5 R2007b (the MathWorks, Natick, MA). Each trial began with the visual presentation of a list of four theme options corresponding to four button press responses (“u,” “i,” “o,” or “p” keyboard buttons). This list remained on the screen for 3 s, the screen blanked for 750 ms, and then the movie played for 10 s. Following the presentation of the movie, the four response options were presented again, and remained on the screen until a response was made. Each unique video was presented twice, resulting in 64 trials total. Video order was randomized for each participant, and the response options for each trial included the intended theme and three randomly selected alternatives.

### Neuroimaging procedure

fMRI data were collected with a Siemens 3.0 T Magnetom Tim Trio system using a 12-channel phased array head coil. Functional images were acquired with a T2^*^ weighted single shot gradient echo, echo-planar sequence sensitive to Blood Oxygen Level Dependent (BOLD) contrast (TR = 2 s; TE = 30 ms; FA = 90°; FOV = 19.2 cm). Each volume consisted of 37 slices acquired parallel to the AC–PC plane (interleaved acquisition; 3 mm thick with 0.5 mm gap; 3 × 3 mm in-plane resolution; 64 × 64 matrix).

Each participant completed four functional scanning runs lasting approximately 7.5 min while viewing danced or acted expressive movement sequences. While there were a total of eight themes in the stimulus set for the study, each scanning run depicted only two of those eight themes. Over the course of all four scanning runs, all eight themes were depicted. Trial sequences were arranged such that theme of a movement sequence was either novel or repeated with respect to the previous trial. This allowed for the analysis of BOLD response RS for repeated vs. novel themes. Each run presented 24 video clips (3 presentations of 8 unique videos depicting 2 themes × 2 dancers × 2 camera angles). Novel and repeated themes were intermixed within each scanning run, with no more than three sequential repetitions of the same theme. Two scanning runs depicted dance and two runs depicted pantomime performances. The order of runs was randomized for each participant. The experiment was controlled using Presentation software (version 13.0, Neurobehavioral Systems Inc, CA). Participants were instructed to focus on the movement performance while viewing the videos. No specific information about the themes portrayed or types of movement used was provided, and no motor responses were required.

### Analysis

For the behavioral data collected outside the scanner, mean consistency scores and mean response time (RT; ms) were computed for each participant. Consistency and RT were each submitted to an ANOVA with Movement Type (dance vs. pantomime) as a within-subjects factor using Stata/IC 10.0 for Macintosh.

Statistical analysis of the neuroimaging data was organized to identify: (1) brain areas responsive to the observation of expressive movement sequences, defined by BOLD activity relative to an implicit baseline, (2) brain areas directly involved in decoding meaning from movement, defined by RS for repeated themes, (3) brain areas in which processes for decoding thematic meaning varied as a function of abstractness, defined by greater RS for danced than pantomimed themes, and (4) the specific pattern of BOLD activity differences for novel and repeated themes as a function of danced or pantomimed movements in regions showing greater RS for dance.

The fMRI data were analyzed using Statistical Parametric Mapping software (SPM5, Wellcome Department of Imaging Neuroscience, London; www.fil.ion.ucl.ac.uk/spm) implemented in Matlab 7.5 R2007b (The MathWorks, Natick, MA). Individual scans were realigned, slice-time corrected and spatially normalized to the Montreal Neurological Institute (MNI) template in SPM5 with a resampled resolution of 3 × 3 × 3 mm. A smoothing kernel of 8 mm was applied to the functional images. A general linear model was created for each participant using SPM5. Parameter estimates of event-related BOLD activity were computed for novel and repeated themes depicted by danced and pantomimed movements, separately for each scanning run, for each participant.

Because the intended theme of each movement sequence was not expressed at a discrete time point but rather throughout the duration of the 10 s video clip, the most appropriate hemodynamic response function (HRF) with which to model the BOLD response at the individual level was determined empirically prior to parameter estimation. Of interest was whether the shape of the BOLD response to these relatively long video clips differed from the canonical HRF typically implemented in SPM. The shape of the BOLD response was estimated for each participant by modeling a finite impulse response function (Ollinger et al., 2001). Each trial was represented by a sequence of 12 consecutive TRs, beginning at the onset of each video clip. Based on this deconvolution, a set of beta weights describing the shape of the response over a 24 s interval was obtained for both novel and repeated themes depicted by both danced and pantomimed movement sequences. To determine whether adjustments should be made to the canonical HRF implemented in SPM, the BOLD responses of a set of 45 brain regions within a known AON were evaluated (see Table 1 for a complete list). To find the most representative shape of the BOLD response within the AON, deconvolved beta weights for each condition were averaged across sessions and collapsed by singular value decomposition analysis (Golub and Reinsch, 1970). This resulted in a characteristic signal shape that maximally described the actual BOLD response in AON regions for both novel and repeated themes, for both danced and pantomimed sequences. This examination of the BOLD response revealed that its time-to-peak was delayed 4 s compared to the canonical HRF response curve typically implemented in SPM. That is, the peak of the BOLD response was reached at 8–10 s following stimulus onset instead of the canonical 4–6 s. Given this result, parameter estimation for conditions of interest in our main analysis was based on a convolution of the design matrix for each participant with a custom HRF that accounted for the observed 4 s delay. Time-to-peak of the HRF was adjusted from 6 to 10 s while keeping the same overall width and height of the canonical function implemented in SPM. Using this custom HRF, the 10 s video duration was modeled as usual in SPM by convolving the HRF with a 10 s boxcar function.

**Table 1:** **The action observation network, as defined by previous investigations**.

**Region**	**Position**	**Structure**	**BA**	**Hemi**.	**Coordinates**			**References**
					**x**	**y**	**z**	
**PREHENSILE ACTION OBSERVATION**								
Superior frontal gyrus	Dorsal	Premotor cortex	6	L	−18	−4	72	Hamilton and Grafton, 2008
			6	R	18	−4	72	Hamilton and Grafton, 2008^**^
Precentral gyrus	Dorsal	Premotor cortex	6	L	−32	−12	69	Buccino et al., 2001
	Dorsolateral	Premotor cortex	6	R	40	−7	65	Buccino et al., 2001
	Middle	Primary motor cortex, Premotor cortex	4a/6	L	−45	−6	48	Buccino et al., 2001
	Ventral	Premotor cortex	6	L	−54	3	26	Buccino et al., 2001^*^
	Ventrolateral	Premotor cortex	6	R	45	−2	48	Buccino et al., 2001
Inferior frontal gyrus	Dorsomedial	Pars opercularis, Broca's area	44	L	−46	4	18	Hamilton and Grafton, 2008
				R	42	12	18	Hamilton and Grafton, 2008
	Lateral	Pars opercularis, Broca's area	44/45	R	57	12	14	Buccino et al., 2001
Superior parietal lobule			7a	L	−27	−66	65	Buccino et al., 2001; Hamilton and Grafton, 2008^*^
	Anterior	Intraparietal sulcus	40/2	L	−36	−44	54	Buccino et al., 2001
				R	40	−44	54	Buccino et al., 2001
			40	R	41	−44	47	Tunik et al., 2007
				L	−40	−42	45	Tunik et al., 2007
	Middle	Intraparietal sulcus	7	L	−32	−56	46	Hamilton and Grafton, 2006
Inferior parietal lobule	Anterior	Supramarginal gyrus/Postcentral sulcus	40/2	L	−56	−26	46	Hamilton and Grafton, 2008
				R	56	−26	46	Hamilton and Grafton, 2008^**^
		Supramarginal gyrus	40/2	R	58	−30	32	Hamilton and Grafton, 2008
			40	L	−58	−34	30	Hamilton and Grafton, 2008
Middle temporal gyrus, Superior temporal sulcus	Posterior	Occipitotemporal, Temporoparietal junction	37/21	L	−50	−62	12	Hamilton and Grafton, 2008
Inferior temporal gyrus	Posterior	Occipitotemporal, V5	37	L	−51	−60	−4	Hamilton and Grafton, 2008^*^
				R	44	−56	−8	Hamilton and Grafton, 2008
Caudate	Posterior	Tail		L	−20	−4	30	Hamilton and Grafton, 2006
Putamen	Anterior			L	−26	10	−6	Hamilton and Grafton, 2006
Cerebellum	Lateral	Crus		L	−50	−56	−36	Hamilton and Grafton, 2006
				R	50	−56	−36	Hamilton and Grafton, 2006^**^
**DANCE OBSERVATION**								
Superior frontal gyrus	Posterior	Dorsal premotor cortex	6	L	−27	−6	72	Calvo-Merino et al., 2005
Precentral sulcus	Middle	Premotor cortex	6	L	−36	0	45	Cross et al., 2006
			6	L	−54	0	45	Calvo-Merino et al., 2005
	Dorsal	Premotor cortex	6	R	30	−6	69	Calvo-Merino et al., 2005
Middle frontal gyrus	Posterior	Premotor cortex	6	R	36	0	45	Cross et al., 2006^**^
Inferior frontal gyrus	Dorsal	Pars opercularis, Broca's area	44	L	−51	9	27	Cross et al., 2006^*^
Superior frontal gyrus/Juxtapositional lobule	Medial	Supplementary Motor Cortex	6	L/R	0	−6	57	Cross et al., 2006
		Pre−supplementary motor cortex	6	L	−3	6	54	Cross et al., 2006
		Pre−supplementary motor cortex	6	R	3	6	54	Cross et al., 2006^**^
Paracingulate gyrus	Medial		6	R	9	12	42	Cross et al., 2006^**^
Postcentral gyrus	Ventral	Primary somatosensory	1	R	64	−16	35	Cross et al., 2006 ^*^
Superior parietal lobule			7/2	L	−33	−45	68	Cross et al., 2006; Hamilton and Grafton, 2008^*^
			7	R	25	−67	63	Cross et al., 2006; Buccino et al., 2001^*^
	Anterior	Intraparietal sulcus	40	L	−33	−45	54	Calvo-Merino et al., 2005
			40	L	−36	−51	36	Cross et al., 2009
		Primary somatosensory	2	R	33	−42	48	Calvo-Merino et al., 2005
Inferior parietal lobule	Posterior	Temporoparietal junction	39/7	L	−39	−66	36	Calvo-Merino et al., 2005
	Ventral	Angular gyrus/Posterior middle temporal gyrus	39/21	R	45	−48	18	Cross et al., 2006

*The first 26 regions were drawn from studies of prehensile reaching and grasping hand movements. The remaining 19 regions listed were drawn from studies of dance observation. Peak voxel coordinates from these studies were used to create 10 mm spherical regions of interest. The time-course of BOLD responses in these AON regions during expressive movement observation was assessed, and provided the basis for determining the most appropriate hemodynamic response function with which to model a whole-brain RS analysis. BA, Brodmann Area; Hemi, Hemisphere; L, left; R, right. MNI coordinates are in millimeters: x = distance right (+) or left (−) to the mid-sagittal plane; y = distance anterior (+) or posterior (−) to vertical plane through anterior commissure; z = distance above (+) or below (−) intercommisural (AC–PC) line*.

“*” *denotes voxel coordinates obtained by averaging peak coordinates from multiple voxels in the same brain region, either within one study or across studies*.

“**” *denotes voxel coordinates determined based on findings showing peak activation in the corresponding voxel in the opposite hemisphere*.

Second-level whole-brain analysis was conducted with SPM8 using a 2 × 2 random effects model with Movement Type and Repetition as within-subject factors using the weighted parameter estimates (contrast images) obtained at the individual level as data. A gray matter mask was applied to whole-brain contrast images prior to second-level analysis to remove white matter voxels from the analysis. Six second-level contrasts were computed, including (1) expressive movement observation (BOLD relative to baseline), (2) dance observation effect (danced sequences > pantomimed sequences), (3) pantomime observation effect (pantomimed sequences > danced sequences), (4) RS (novel themes > repeated themes), (5) dance × repetition interaction (RS for dance > RS for pantomime), and (6) pantomime x repetition interaction (RS for pantomime > RS for dance). Following the creation of T-map images in SPM8, FSL was used to create Z-map images (Version 4.1.1; Analysis Group, FMRIB, Oxford, UK; Smith et al., 2004; Jenkinson et al., 2012). The results were thresholded at *p* < 0.05, cluster-corrected using FSL subroutines based on Gaussian random field theory (Poldrack et al., 2011; Nichols, 2012). To examine the nature of the differences in RS between dance and pantomime, a mask image was created based on the corresponding cluster-thresholded Z-map of regions showing greater RS for dance, and the mean BOLD activity (contrast image values) was computed for novel and repeated dance and pantomime contrasts from each participant's first-level analysis. Mean BOLD activity measures were submitted to a 2 × 2 ANOVA with Movement Type (dance vs. pantomime) and Repetition (novel vs. repeat) as within-subjects factors using Stata/IC 10.0 for Macintosh.

In order to ensure that observed RS effects for repeated themes were not due to low-level kinematic effects, a motion tracking analysis of all 32 videos was performed using Tracker 4.87 software for Mac (written by Douglas Brown, distributed on the Open Source Physics platform, www.opensourcephysics.org). A variety of motion parameters, including velocity, acceleration, momentum, and kinetic energy, were computed within the Tracker software based on semi-automated/supervised motion tracking of the top of the head, one hand, and one foot of each performer. The key question relevant to our results was whether there was a difference in motion between videos depicting novel and repeated themes. One factor ANOVAs for each motion parameter revealed no significant differences in coarse kinematic profiles between “novel” and “repeated” theme trials (all *p*'s > 0.05). This was not particularly surprising given that all videos were used for both novel and repeated themes, which were defined entirely based on trial sequence). In contrast, the comparison between danced and pantomimed themes did reveal significant differences in kinematic profiles. A 2 × 3 ANOVA with Movement Type (Dance, Pantomime) and Body Point (Hand, Head, Foot) as factors was conducted for each motion parameter (velocity, acceleration, momentum, and kinetic energy), and revealed greater motion energy on all parameters for the danced themes compared to the pantomimed themes (all *p*'s < 0.05). Any differences in RS between danced and pantomimed themes may therefore be attributed to differences in kinematic properties of body movement. Importantly, however, because there were no systematic differences in motion kinematics between novel and repeated themes, any RS effects for repeated themes could not be attributed to the effect of motion kinematics.

## Results

**Figure 3:**
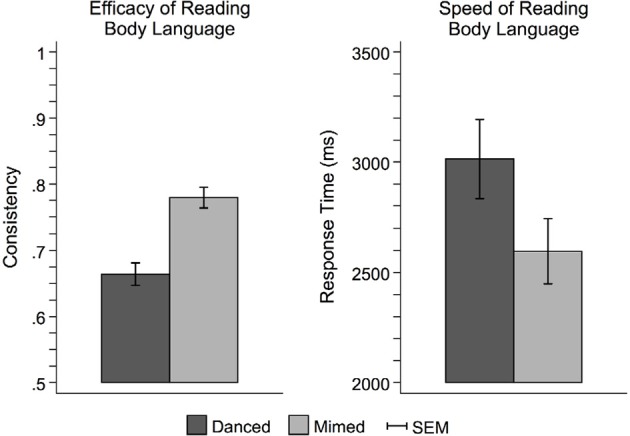
**Behavioral performance on the theme judgment task**. Participants more readily interpreted pantomime than dance. This was evidenced by both greater consistency between the meaningful theme intended to be expressed by the performer and the interpretive judgments made by the observer (left), and faster response times (right). This pattern of results suggests that dance was more difficult to interpret than pantomime, perhaps owing to the use of more abstract metaphors to link movement with meaning. Pantomime, on the other hand, relied on more concrete, mundane sorts of movements that were more likely to carry meaningful associations based on observers' prior everyday experience. SEM, standard error of the mean.

Figure 3 illustrates the behavioral results of the interpretation task completed outside the scanner. Participants had higher consistency scores for pantomimed movements than danced movements [*F*_(1, 42)_ = 42.06, *p* < 0.0001], indicating better transmission of the intended expressive meaning from performer to viewer. Pantomimed sequences were also interpreted more quickly than danced sequences [*F*_(1, 42)_ = 27.28, *p* < 0.0001], suggesting an overall performance advantage for pantomimed sequences.

### Expressive whole-body movements engage the action observation network

**Figure 4:**
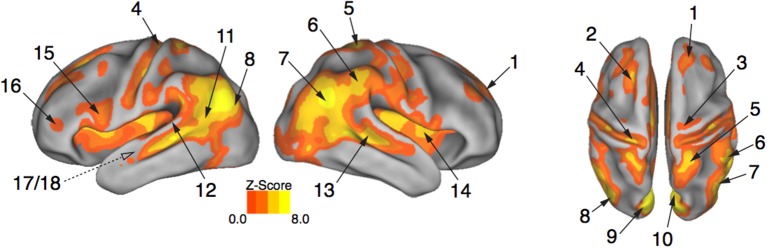
**Expressive performances engage the action observation network**. Viewing expressive whole-body movement sequences engaged a distributed cortical action observation network (*p* < 0.05, FWE corrected). Large areas of parietal, temporal, frontal, and insular cortex included somatosensory, motor, and premotor regions that have been considered previously to comprise a human “mirror neuron” system, as well as non-motor areas linked to comprehension, social perception, and affective decision-making. Number labels correspond to those listed in Table 2, which provides anatomical names and voxel coordinates for areas of peak activation. Dotted line for regions 17/18 indicates medial temporal position not visible on the cortical surface.

Brain activity associated with the observation of expressive movement sequences was revealed by significant BOLD responses to observing both dance and pantomime movement sequences, relative to the inter-trial resting baseline. Figure 4 depicts significant activation (*p* < 0.05, cluster corrected in FSL) rendered on an inflated cortical surface of the Human PALS-B12 Atlas (Van Essen, 2005) using Caret (Version 5. 61; http://www.nitrc.org/projects/caret; Van Essen et al., 2001). Table 2 presents the MNI coordinates for selected voxels within clusters active during movement observation, as labeled in Figure 4. Region names were obtained from the Harvard-Oxford Cortical and Subcortical Structural Atlases (Frazier et al., 2005; Desikan et al., 2006; Makris et al., 2006; Goldstein et al., 2007; Harvard Center for Morphometric Analysis; www.partners.org/researchcores/imaging/morphology_MGH.asp), and Brodmann Area labels were obtained from the Juelich Histological Atlas (Eickhoff et al., 2005, 2006, 2007), as implemented in FSL. Observation of body movement was associated with robust BOLD activation encompassing cortex typically associated with the AON, including fronto-parietal regions linked to the representation of action kinematics, goals, and outcomes (Hamilton and Grafton, 2006, 2007), as well as temporal, occipital, and insular cortex and subcortical regions including amygdala and hippocampus—regions typically associated with language comprehension (Kirchhoff et al., 2000; Ni et al., 2000; Friederici et al., 2003) and socio-affective information processing and decision-making (Anderson et al., 1999; Adolphs et al., 2003; Bechara et al., 2003; Bechara and Damasio, 2005).

**Table 2:** **Brain regions showing a significant BOLD response while participants viewed expressive whole-body movement sequences**.

**Label**	**Region**	**Position**	**Structure**	**BA**	**Hemi**.	**Coordinates**			**Z Score**
						**x**	**y**	**z**	
1	Superior frontal gyrus	Anterior	Frontal Pole	10	L	−6	56	20	6.05
				10	R	8	56	16	3.64
2		Dorsal		6	L	−8	14	64	4.27
				6	R	14	16	64	3.72
3		Medial	Supplementary motor	6	L	−6	−16	48	4.55
			Area	6	R	8	−16	50	4.90
4	Postcentral gyrus	Dorsal	Primary somatosensory	3/4	L	−22	−34	62	5.14
			Cortex	3/4	R	20	−34	64	4.95
5	Superior parietal lobule	Dorsal		5	L	−14	−56	70	4.73
				5	R	12	−52	70	5.26
6		Anterior	Supramarginal Gyrus	40/48	L	−58	−44	32	4.64
				40	R	60	−44	34	4.76
7	Inferior parietal lobule	Posterior	Angular gyrus	39	L	−56	−56	26	5.68
				39/40	R	54	−52	30	4.58
8	Lateral occipital cortex	Superior		39	L	−48	−64	32	6.73
				39	R	48	−64	32	6.42
9	Lingual gyrus	Inferior	V2	18	L	−12	−64	2	9.15
				18	R	12	−64	2	9.27
10	Intracalcarine cortex	Inferior	V1	17	L	−14	−76	6	9.39
				17	R	14	−76	6	11.47
11	Middle temporal gyrus	Posterior		21	L	−50	−52	4	5.10
				21	R	56	−50	4	6.11
12	Planum temporale	Posterior		22	L	−56	−20	6	4.38
				22	R	60	−18	6	3.96
13	Superior temporal gyrus	Posterior		21/22	L	−50	−20	−6	5.58
				21/22	R	58	−20	2	5.09
14	Insular cortex	Posterior		48	L	−32	−22	10	5.58
				48	R	36	−18	18	5.46
15	Central operculuar cortex		Secondary somatosensory	48	L	−42	−18	18	4.68
			Cortex	48	R	44	−12	13	5.15
16	Inferior frontal gyrus	Lateral	Pars opercularis, Broca's area	44	L	−46	14	8	5.63
				44	R	52	10	4	4.21
17	Amygdala	Laterobasal			L	−24	−8	−14	6.83
					R	28	−8	−14	7.54
18	Hippocampus	Medial	Dentate gyrus	20/48	L	−30	−28	−12	4.28
				48	R	30	−22	12	5.43

*BOLD activations (p < 0.05, corrected FWE) were distributed throughout the AON. Voxel coordinates listed were determined by visual inspection of peak activity in selected clusters. “Label” column refers to the corresponding brain region highlighted in Figure 4. BA, Brodmann Area; Hemi, Hemisphere; L, left; R, right. MNI coordinates are in millimeters: x = distance right (+) or left (−) to the mid-sagittal plane; y = distance anterior (+) or posterior (−) to vertical plane through anterior commissure; z = distance above (+) or below (−) intercommisural (AC–PC) line*.

### The action observation network “reads” body language

**Figure 5:**
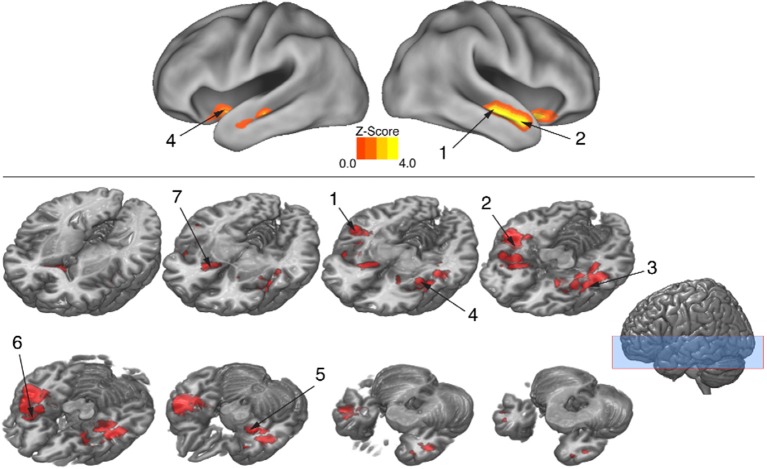
**BOLD suppression (RS) reveals brain substrates for “reading” body language**. Regions involved in decoding meaning in body language showing were isolated by testing for BOLD suppression when the intended theme of an expressive performance was repeated across trials. To identify regions showing RS, BOLD activity associated with novel themes was contrasted with BOLD activity associated with repeated themes (*p* < 0.05, cluster corrected in FSL). Significantly greater activity for novel relative to repeated themes was evidence of RS. Given that the intended theme of a performance was the only element that was repeated between trials, regions showing RS revealed brain substrates that were sensitive to the specific meaning infused into a movement sequence by a performer. Number labels correspond to those listed in Table 3, which provides anatomical names and voxel coordinates for key clusters showing significant RS. Blue shaded area indicates vertical extent of axial slices shown.

To isolate brain areas that decipher meaning conveyed by expressive body movement, regions showing RS (reduced BOLD activity for repeated compared to novel themes) were identified. Since theme was the only stimulus dimension repeated systematically across trials for this comparison, decreased activation for repeated themes could not be attributed to physical features of the stimulus such as particular movements, performers, or camera viewpoints. Figure 5 illustrates brain areas showing significant suppression for repeated themes (*p* < 0.05, cluster corrected in FSL). Table 3 presents the MNI coordinates for selected voxels within significant clusters. RS was found bilaterally on the rostral bank of the middle temporal gyrus extending into temporal pole and orbitofrontal cortex. There was also significant suppression in bilateral amygdala and insular cortex.

**Table 3:** **Brain regions showing significant BOLD suppression for repeated themes (***p*** < 0.05, cluster corrected in FSL)**.

**Label**	**Region**	**Location**	**Structure**	**BA**	**Hemi**.	**Coordinates**			**Z Score**
						**x**	**y**	**z**	
1	Middle temporal gyrus	Middle	STS	20/21	L	−52	−16	−12	3.07
				20/21	R	56	−14	−12	3.31
2		Anterior	STS	20/21	L	−50	−2	−26	3.40
				20/21	R	50	−2	−26	3.45
3	Temporal pole	Anterior		21/38	L	−48	0	−10	2.94
				21/38	R	44	14	−20	2.64
4	Insular cortex	Anterior		48	L	−40	6	−10	3.01
				48	R	34	8	−14	3.80
5	Amygdala	Laterobasal			L	−26	−6	−22	2.43
					R	30	−6	−22	4.70
6	Orbitofrontal cortex	Ventrolateral		38/47	L	−34	16	−18	2.69
				38/47	R	30	20	−18	3.37
7	Orbitofrontal cortex/putamen	Ventromedial		11	L	−18	14	−8	2.55
				11	R	20	14	−10	3.51

*Voxel coordinates listed were determined by visual inspection of peak activity in selected clusters. “Label” column refers to the corresponding brain region highlighted in Figure 5. BA, Brodmann area; Hemi, Hemisphere; L, left; R, right. MNI coordinates are in millimeters: x = distance right (+) or left (−) to the mid-sagittal plane; y = distance anterior (+) or posterior (−) to vertical plane through anterior commissure; z = distance above (+) or below (−) intercommisural (AC–PC) line*.

### Movement abstractness challenges brain substrates that decode meaning

**Figure 6:**
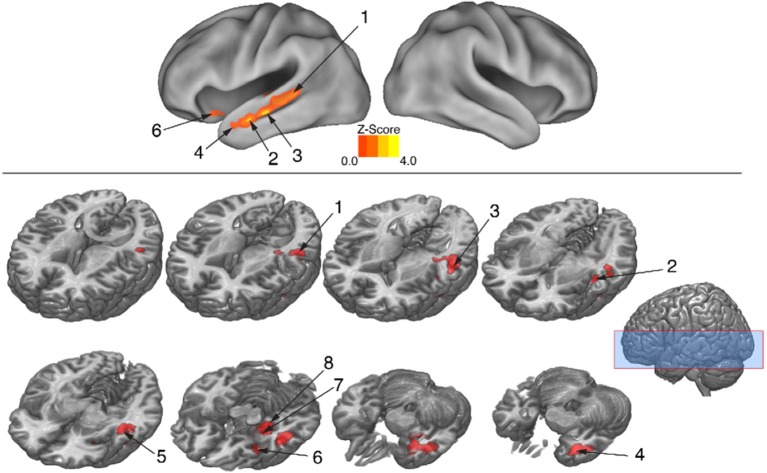
**Regions showing greater RS for dance than pantomime**. RS effects were compared between movement types. This was implemented as an interaction contrast within our Movement Type × Repetition ANOVA design [(Novel Dance > Repeated Dance) > (Novel Pantomime > Repeated Pantomime)]. Greater RS for dance was lateralized to left hemisphere meaning-sensitive regions. The brain areas shown here have been linked previously to the comprehension of meaning in verbal language, suggesting the possibility they represent shared brain substrates for building meaning from both language and action. Number labels correspond to those listed in Table 4, which provides anatomical names and voxel coordinates for key clusters showing significantly greater RS for dance. Blue shaded area indicates vertical extent of axial slices shown.

The behavioral analysis indicated that interpreting danced themes was more difficult than interpreting pantomimed themes, as evidenced by lower consistency scores and greater RTs. Previous research indicates that greater difficulty discriminating a particular stimulus dimension is associated with greater BOLD suppression upon repetition of that dimension's attributes (Hasson et al., 2006). To test whether greater difficulty decoding meaning from dance than pantomime would also be associated with greater RS in the present data, the magnitude of BOLD response suppression was compared between movement types. This was done with the Dance × Repetition interaction contrast in the second-level whole brain analysis, which revealed regions that had greater RS for dance than for pantomime. Figure 6 illustrates brain regions showing greater RS for themes portrayed through dance than pantomime (*p* < 0.05, cluster corrected in FSL). Significant differences were entirely left-lateralized in superior and middle temporal gyri, extending into temporal pole and orbitofrontal cortex, and also present in laterobasal amygdala and the cornu ammonis of the hippocampus. Table 4 presents the MNI coordinates for selected voxels within significant clusters. The reverse Pantomime × Repetition interaction was also tested, but did not reveal any regions showing greater RS for pantomime than dance (*p* > 0.05, cluster corrected in FSL).

**Table 4:** **Brain regions showing significantly greater RS for themes expressed through dance relative to themes expressed through pantomime (***p*** < 0.05, cluster corrected in FSL)**.

**Label**	**Region**	**Location**	**Structure**	**BA**	**Hemi**.	**Coordinates**			**Z Score**
						**x**	**y**	**z**	
1	Inferior parietal lobule	Posterior	Angular gyrus	21/22	L	−50	−44	12	2.60
2	Superior temporal gyrus	Anterior		21/22	L	−52	−6	−12	3.22
3		Posterior		21/22	L	−52	−32	2	2.89
4	Middle temporal gyrus	Anterior		20/21	L	−50	0	−26	2.91
5	Temporal pole			20/38	L	−40	14	−26	2.54
6	Orbitofrontal cortex			38	L	−38	18	−18	2.68
7	Amygdala	Laterobasal			L	−26	−6	−18	2.77
8	Hippocampus		Cornu ammonis	20	L	−28	−16	−18	3.27

*Voxel coordinates listed were determined by visual inspection of peak activity in selected clusters. “Label” column refers to the corresponding brain region highlighted in Figure 6. BA, Brodmann Area; Hemi, hemisphere; L, left; R, right. MNI coordinates are in millimeters: x = distance right (+) or left (−) to the mid-sagittal plane; y = distance anterior (+) or posterior (−) to vertical plane through anterior commissure; z = distance above (+) or below (−) intercommisural (AC–PC) line*.

**Figure 7:**
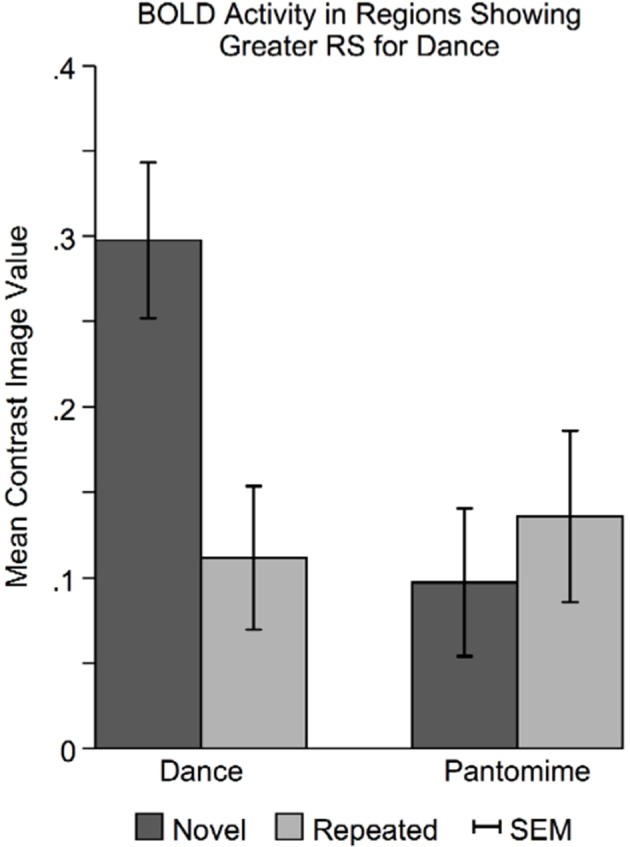
**Novel danced themes challenge brain substrates that decode meaning from movement**. To determine the specific pattern of BOLD activity that resulted in greater RS for dance, average BOLD activity in these areas was computed for each condition separately. Greater RS for dance was driven by a larger BOLD response to novel danced themes. Considered together with behavioral findings indicating that dance was more difficult to interpret, greater RS for dance seems to result from a greater processing “challenge” to brain substrates involved in decoding meaning from movement. SEM, standard error of the mean.

In regions showing greater RS for dance than pantomime, mean BOLD responses for novel and repeated dance and pantomime conditions were computed across voxels for each participant based on their first-level contrast images. This was done to test whether the greater RS for dance was due to greater activity in the novel condition, lower activity in the repeated condition, or some combination of both. Figure 7 illustrates a pattern of BOLD activity across conditions demonstrates that the greater RS for dance was the result of greater initial BOLD activation in response to novel themes. The ANOVA results showed a significant Movement Type × Repetition interaction [*F*_(1, 42)_ = 7.83, *p* < 0.01], indicating that BOLD activity in response to novel danced themes was greater than BOLD activity for all other conditions in these regions.

## Discussion

This study was designed to reveal brain regions involved in reading body language—the meaningful information we pick up about the affective states and intentions of others based on their body movement. Brain regions that decoded meaning from body movement were identified with a whole brain analysis of RS that compared BOLD activity for novel and repeated themes expressed through modern dance or pantomime. Significant RS for repeated themes was observed bilaterally, extending anteriorly along middle and superior temporal gyri into temporal pole, medially into insula, rostrally into inferior orbitofrontal cortex, and caudally into hippocampus and amygdala. Together, these brain substrates comprise a functional system within the larger AON. This suggests strongly that decoding meaning from expressive body movement constitutes a dimension of action representation not previously isolated in studies of action understanding. In the following we argue that this embedding is consistent with the hierarchical organization of the AON.

### Body language as superordinate in a hierarchical action observation network

Previous investigations of action understanding have identified the AON as a key a cognitive system for the organization of action in general, highlighting the fact that both performing and observing action rely on many of the same brain substrates (Grafton, 2009; Ortigue et al., 2010; Kilner, 2011; Ogawa and Inui, 2011; Uithol et al., 2011; Grafton and Tipper, 2012). Shared brain substrates for controlling one's own action and understanding the actions of others are often taken as evidence of a “mirror neuron system” (MNS), following from physiological studies showing that cells in area F5 of the macaque monkey premotor cortex fired in response to both performing and observing goal-directed actions (Pellegrino et al., 1992; Gallese et al., 1996; Rizzolatti et al., 1996a). Since these initial observations were made regarding monkeys, there has been a tremendous effort to characterize a human analog of the MNS, and incorporate it into theories of not only action understanding, but also social cognition, language development, empathy, and neuropsychiatric disorders in which these faculties are compromised (Gallese and Goldman, 1998; Rizzolatti and Arbib, 1998; Rizzolatti et al., 2001; Gallese, 2003; Gallese et al., 2004; Rizzolatti and Craighero, 2004; Iacoboni et al., 2005; Tettamanti et al., 2005; Dapretto et al., 2006; Iacoboni and Dapretto, 2006; Shapiro, 2008; Decety and Ickes, 2011). A fundamental assumption common to all such theories is that mirror neurons provide a direct neural mechanism for action understanding through “motor resonance,” or the simulation of one's own motor programs for an observed action (Jacob, 2008; Oosterhof et al., 2013). One proposed mechanism for action understanding through motor resonance is the embodiment of sensorimotor associations between action goals and specific motor behaviors (Mitz et al., 1991; Niedenthal et al., 2005; McCall et al., 2012). While the involvement of the motor system in a range of social, cognitive and affective domains is certainly worthy of focused investigation, and mirror neurons may well play an important role in supporting such “embodied cognition,” this by no means implies that mirror neurons alone can account for the ability to garner meaning from observed body movement.

Since the AON is a distributed cortical network that extends beyond motor-related brain substrates engaged during action observation, it is best characterized not as a homogeneous “mirroring” mechanism, but rather as a collection of functionally specific but interconnected modules that represent distinct properties of observed actions (Grafton, 2009; Grafton and Tipper, 2012). The present results build on this functional-hierarchical model of the AON by incorporating meaningful expression as an inherent aspect of body movement that is decoded in distinct regions of the AON. In other words, the bilateral temporal-orbitofrontal regions that showed RS for repeated themes comprise a distinct functional module of the AON that supports an additional level of the action representation hierarchy. Such an interpretation is consistent with the idea that action representation is inherently nested, carried out within a hierarchy of part-whole processes for which higher levels depend on lower levels (Cooper and Shallice, 2006; Botvinick, 2008; Grafton and Tipper, 2012). We propose that the meaning infused into the body movement of a person having a particular affective stance is decoded superordinately to more concrete properties of action, such as kinematics and object goals. Under this view, while decoding these representationally subordinate properties of action may involve motor-related brain substrates, decoding “body language” engages non-motor regions of the AON that link movement and meaning, relying on inputs from lower levels of the action representation hierarchy that provide information about movement kinematics, prosodic nuances, and dynamic inflections.

While the present results suggest that the temporal-orbitofrontal regions identified here as decoding meaning from emotive body movement constitute a distinct functional module within a hierarchically organized AON, it is important to note that these regions have not previously been included in anatomical descriptions of the AON. The present study, however, isolated a property of action representation that had not been previously investigated; so identifying regions of the AON not previously included in its functional-anatomic definition is perhaps not surprising. This underscores the important point that the AON is functionally defined, such that its apparent anatomical extent in a given experimental context depends upon the particular aspects of action representation that are engaged and isolable. Previous studies of another abstract property of action representation, namely intention understanding, also illustrate this point. Inferring the intentions of an actor engages medial prefrontal cortex, bilateral posterior superior temporal sulcus, and left temporo-parietal junction—non-motor regions of the brain typically associated with “mentalizing,” or thinking about the mental states of another agent (Ansuini et al., 2015; Ciaramidaro et al., 2014). A key finding of this research is that intention understanding depends fundamentally on the integration of motor-related (“mirroring”) brain regions and non-motor (“mentalizing”) brain regions (Becchio et al., 2012). The present results parallel this finding, and point to the idea that in the context of action representation, motor and non-motor brain areas are not two separate brain networks, but rather one integrated functional system.

### Predictive coding and the construction of meaning in the action observation network

A critical question raised by the idea that the temporal-orbitofrontal brain regions in which RS was observed here constitute a superordinate, meaning-sensitive functional module of the AON is how activity in subordinate AON modules is integrated at this higher level to produce differential neural firing patterns in response to different meaningful body expressions. That is, what are the neural mechanisms underlying the observed sensitivity to meaning in body language, and furthermore, why are these mechanisms subject to adaptation through repetition (RS)? While the present results do not provide direct evidence to answer these questions, we propose that a “predictive coding” interpretation provides a coherent model of action representation (Brass et al., 2007; Kilner and Frith, 2008; Brown and Brüne, 2012) that yields useful predictions about the neural processes by which meaning is decoded that would account for the observed RS effect. The primary mechanism invoked by a predictive coding framework of action understanding is recurrent feed-forward and feedback processing across the various levels of the AON, which supports a Bayesian system of predictive neural coding, feedback processes, and prediction error reduction at each level of action representation (Friston et al., 2011). According to this model, each level of the action observation hierarchy generates predictions to anticipate neural activity at lower levels of the hierarchy. Predictions in the form of neural codes are sent to lower levels through feedback connections, and compared with actual subordinate neural representations. Any discrepancy between neural predictions and actual representations are coded as prediction error. Information regarding prediction error is sent through recurrent feed-forward projections to superordinate regions, and used to update predictive priors such that subsequent prediction error is minimized. Together, these Bayes-optimal neural ensemble operations converge on the most probable inference for representation at the superordinate level (Friston et al., 2011) and, ultimately, action understanding based on the integration of representations at each level of the action observation hierarchy (Chambon et al., 2011; Kilner, 2011).

A predictive coding account of the present results would suggest that initial feed-forward inputs from subordinate levels of the AON provided the superordinate temporal-orbitofrontal module with information regarding movement kinematics, prosody, gestural elements, and dynamic inflections, which, when integrated with other inputs based on prior experience, would provide a basis for an initial prediction about potential meanings of a body expression. This prediction would yield a generative neural model about the movement dynamics that would be expected given the predicted meaning of the observed body expression, which would be fed back to lower levels of the network that coded movement dynamics and sensorimotor associations. Predictive activity would be contrasted with actual representations as movement information was accrued throughout the performance, and the resulting prediction error would be utilized via feed-forward projections to temporal-orbitofrontal regions to update predictive codes regarding meaning and minimize subsequent prediction error. In this way, the meaningful affective theme being expressed by the performer would be converged upon through recurrent Bayes-optimal neural ensemble operations. Thus, meaning expressed through body language would be accrued iteratively in temporal-orbitofrontal regions by integrating neural representations of various facets of action decoded throughout the AON. Interestingly, and consistent with a model in which an iterative process accrued information over time, we observed that BOLD responses in AON regions peaked more slowly than expected based on SPM's canonical HRF as the videos were viewed over an extended (10 s) duration. Under an iterative predictive coding model, RS for repeated themes could be accounted for by reduced initial generative activity in temporal-orbitofrontal regions due to better constrained predictions about potential meanings conveyed by observed movement, more efficient convergence on an inference due to faster minimization of prediction error, or some combination of both of these mechanisms. The present results provide indirect evidence for the former account, in that more abstract, less constrained movement metaphors relied upon by expressive dance resulted in greater RS due to larger BOLD responses for novel themes relative to the more concrete, better-constrained associations conveyed by pantomime.

### Shared brain substrates for meaning in action and language

The middle temporal gyrus and superior temporal sulcus regions identified here as part of a functional module of the AON that “reads” body language have been linked previously to a variety of high-level linguistic domains related to understanding meaning. Among these are conceptual knowledge (Lambon Ralph et al., 2009), language comprehension (Hasson et al., 2006; Noppeney and Penny, 2006; Price, 2010), sensitivity to the congruency between intentions and actions, both verbal/conceptual (Deen and McCarthy, 2010), and perceptual/implicit (Wyk et al., 2009), as well as understanding abstract language and metaphorical descriptions of action (Desai et al., 2011). While together these studies demonstrate that high-level linguistic processing involves bilateral superior and middle temporal regions, there is evidence for a general predominance of the left hemisphere in comprehending semantics (Price, 2010), and a predominance of the right hemisphere in incorporating socio-emotional information and affective context (Wyk et al., 2009). For example, brain atrophy associated with a primary progressive aphasia characterized by profound disturbances in semantic comprehension occurs bilaterally in anterior middle temporal regions, but is more pronounced in the left hemisphere (Gorno-Tempini et al., 2004). In contrast, neural degeneration in right hemisphere orbitofrontal, insula, and anterior middle temporal regions is associated not only with semantic dementia but also deficits in socio-emotional sensitivity and regulation (Rosen et al., 2005).

This hemispheric asymmetry in brain substrates associated with interpreting meaning in verbal language is paralleled in the present results, which not only link the same bilateral temporal-orbitofrontal brain substrates to comprehending meaning from affectively expressive body language, but also demonstrate a predominance of the left hemisphere in deciphering the particularly abstract movement metaphors conveyed by dance. This asymmetry was evident as greater RS for repeated themes for dance relative to pantomime, which was driven by a greater initial activation for novel themes, suggesting that these left-hemisphere regions were engaged more vigorously when decoding more abstract movement metaphors. Together, these results illustrate a striking overlap in the brain substrates involved in processing meaning in verbal language and decoding meaning from expressive body movement. This overlap suggests that a long-hypothesized evolutionary link between gestural body movement and language (Hewes et al., 1973; Harnad et al., 1976; Rizzolatti and Arbib, 1998; Corballis, 2003) may be instantiated by a network of shared brain substrates for representing semiotic structure, which constitutes the informational scaffolding for building meaning in both language and gesture (Lemke, 1987; Freeman, 1997; McNeill, 2012; Lhommet and Marsella, 2013). While speculative, under this view the temporal-orbitofrontal AON module for coding meaning observed may provide a neural basis for semiosis (the construction of meaning), which would lend support to the intriguing philosophical argument that meaning is fundamentally grounded in processes of the body, brain, and the social environment within which they are immersed (Thibault, 2004).

### Summary and conclusions

The present results identify a system of temporal, orbitofrontal, insula, and amygdala brain regions that supports the meaningful interpretation of expressive body language. We propose that these areas reveal a previously undefined superordinate functional module within a known, stratified hierarchical brain network for action representation. The findings are consistent with a predictive coding model of action understanding, wherein the meaning that is imbued into expressive body movements through subtle kinematics and prosodic nuances is decoded as a distinct property of action via feed-forward and feedback processing across the levels of a hierarchical AON. Under this view, recurrent processing loops integrate lower-level representations of movement dynamics and socio-affective perceptual information to generate, evaluate, and update predictive inferences about expressive content that are mediated in a superordinate temporal-orbitofrontal module of the AON. Thus, while lower-level action representation in motor-related brain areas (sometimes referred to as a human “mirror neuron system”) may be a key step in the construction of meaning from movement, it is not these motor areas that code the specific meaning of an expressive body movement. Rather, we have demonstrated an additional level of the cortical action representation hierarchy in non-motor regions of the AON. The results highlight an important link between action representation and language, and point to the possibility of shared brain substrates for constructing meaning in both domains.

## Author contributions

CT, GS, and SG designed the experiment. CT and GS created stimuli, which included recruiting professional dancers and filming expressive movement sequences. GS carried out video editing. CT completed computer programming for experimental control and data analysis. GS and CT recruited participants and conducted behavioral and fMRI testing. CT and SG designed the data analysis and CT and GS carried it out. GS conducted a literature review, and CT wrote the paper with reviews and edits from SG.

### Conflict of interest statement

The authors declare that the research was conducted in the absence of any commercial or financial relationships that could be construed as a potential conflict of interest.
